# Exploring the feasibility and acceptability of integrating screening for gender-based violence into HIV counselling and testing for adolescent girls and young women in Tanzania and South Africa

**DOI:** 10.1186/s12889-021-10454-z

**Published:** 2021-03-03

**Authors:** Manuela Colombini, Fiona Scorgie, Anne Stangl, Sheila Harvey, Lethabo Ramskin, Nomhle Khoza, Emma Mashauri, Deborah Baron, Shelley Lees, Saidi Kapiga, Charlotte Watts, Sinead Delany-Moretlwe, on behalf of the EMPOWER study team

**Affiliations:** 1 Department of Global Health and Development, London School of Hygiene and Tropical Medicine, 15-17 Tavistock Place, London, WC1H 9SH UK; 2 Wits Reproductive Health Institute, Witwatersrand University, Johannesburg, South Africa; 3 International Center for Research on Women, Washington, DC USA; 4 Mwanza Intervention Trials Unit, Mwanza, Tanzania; 5 Department of Infectious Diseases Epidemiology, London School of Hygiene and Tropical Medicine, London, UK

**Keywords:** Gender-based violence, Screening, PrEP, HIV prevention, Adolescent girls and young women, Service integration

## Abstract

**Background:**

Gender-based violence (GBV) undermines HIV prevention and treatment cascades, particularly among women who report partner violence. Screening for violence during HIV testing, and prior to offering pre-exposure prophylaxis (PrEP) to HIV uninfected women, provides an opportunity to identify those at heightened HIV risk and greater potential for non-adherence or early discontinuation of PrEP. The paper describes our experience with offering integrated GBV screening and referral as part of HIV counselling and testing. This component was implemented within EMPOWER, a demonstration project offering combination HIV prevention, including daily oral PrEP, to young women in South Africa and Tanzania.

**Methods:**

Between February 2017 and March 2018, a process evaluation was conducted to explore views, experiences and practices of stakeholders (study participants and study clinical staff) during implementation of the GBV screening component. This article assesses the feasibility and acceptability of the approach from multiple stakeholder perspectives, drawing on counselling session observations (*n* = 10), in-depth interviews with participants aged 16–24 (*n* = 39) and clinical staff (*n* = 13), and notes from debriefings with counsellors. Study process data were also collected (e.g. number of women screened and referred). Following a thematic inductive approach, qualitative data were analysed using qualitative software (NVivo 11).

**Results:**

Findings show that 31% of young women screened positive for GBV and only 10% requested referrals. Overall, study participants accessing PrEP were amenable to being asked about violence during HIV risk assessment, as this offered the opportunity to find emotional relief and seek help, although a few found this traumatic. In both sites, the sensitive and empathetic approach of the staff helped mitigate distress of GBV disclosure. In general, the delivery of GBV screening in HCT proved to be feasible, provided that the basic principles of confidentiality, staff empathy, and absence of judgment were observed. However, uptake of linkage to further care remained low in both sites.

**Conclusion:**

Most stakeholders found GBV screening acceptable and feasible. Key principles that should be in place for young women to be asked safely about GBV during HIV counselling and testing included respect for confidentiality, a youth-friendly and non-judgmental environment, and a functioning referral network.

## Background

Gender-based violence (GBV) is a global public health concern and a human rights violation [1, 2]. Overall, 30% of women worldwide have experienced either physical or sexual intimate partner violence (IPV) or non-partner sexual violence [1]. In Africa, 36.6% of women have experienced violence in their lives, with adolescent girls and young women (AGYW) being at high risk of IPV [1, 3]. Exposure to GBV is associated with long-term health consequences [2, 4], including HIV [5, 6]. Longitudinal studies have shown that GBV increases HIV acquisition for women [5, 7–10], especially for adolescent girls, who are at risk of transactional or forced sex [11–13]. GBV also undermines HIV prevention and treatment cascades [14–21]. Although findings are mixed, studies indicate that women’s fear of IPV prevents them from attending HIV counselling and testing (HCT) services, and could affect uptake, adherence or early discontinuation of PrEP [20], while others have found that IPV is associated with poorer ART adherence [14].

Despite the high prevalence and its adverse health effects, to date the health sector has had limited investments in addressing GBV [22]. GBV screening in health care settings is not routinely recommended owing to insufficient evidence on improved outcomes for women [23] and to potential harm when no staff capacity or referral are available [24]. However, studies have shown that it is acceptable to women [25], and can identify those at risk of violence [26]. Most GBV screening interventions (primarily focusing on partner violence) have been tested in antenatal care [27, 28] and primary health care [29, 30], while only a few studies have integrated GBV screening within HIV services [31–34]. While suggesting that violence identification is feasible and acceptable within HIV counselling services, research findings from Southern Africa highlight barriers related to individual providers (e.g. sporadic implementation due to lack of time) and health facility conditions (e.g. limited referral services) that could affect its implementation by HIV providers [31, 34]. A recent systematic review exploring the impact of IPV on women’s PrEP acceptability and use found scarce research on integration of GBV issues and context within HIV prevention counselling services [35]. Recent studies aiming to reduce new HIV infections among AGYW shifted their focus on minimising critical vulnerabilities such as GBV [36] and reiterated the need for prevention strategies that address violent behaviour or support young women in the removal from their violent situation [37, 38]. However, to our knowledge, only one pilot intervention study (CHARISMA) – currently in its design phase and designed to increase male partner support for female-initiated HIV prevention intervention use - developed a screening tool for partner violence and women’s safety in the context of PreP delivery [39]. Though no acceptability and feasibility data are yet available for the CHARISMA study. Research remains particularly scarce on GBV response strategies that are acceptable for young women and clinical staff in HIV prevention services. Considering the limited evidence on the effects of GBV on PrEP uptake and engagement, there is a need to understand if and how GBV enquiry within PrEP services could enable identification of those with a greater potential for non-adherence or early discontinuation of PrEP due to experiences or fear of GBV. Our paper attempts to fill this evidence gap. This article aims to describe views and experiences with implementing GBV screening and referrals within HCT in a larger PrEP demonstration project (EMPOWER) that sought to assess the acceptability and feasibility of a package of prevention interventions, including oral PrEP, in South Africa (hereafter SA) and Tanzania (hereafter Tz). EMPOWER was implemented within the context of adolescent and youth-friendly services (AYFS).

## Methods

### Brief description of GBV screening and referral component

Our definition of GBV encompassed any violence (physical, emotional, sexual and economic) perpetrated by sexual partners, family members, peers or strangers. The rationale for using a broad definition in developing the GBV screening component is based on evidence showing that AGYW experience both partner and non-partner violence [11].

The GBV screening component included various activities (see Table 1 for more information). Before implementing the screening, five study clinical staff (two HIV counsellors in SA, and three clinical staff in Tz) were trained on GBV identification by a GBV researcher. Regular staff supervision was also provided to monitor study fidelity.

**Table 1:** GBV screening and linkage to care component: activities/inputs

Description	Implementation
Activities/inputs included: - Training for study clinical staff offering HCT - GBV screening and risk assessment job aid for study clinical staff - Safety planning job aid - Material on GBV referral services for staff and participants - Staff supervision though debriefing sessions	- Prior to study implementation, clinical staff offering GBV screening in HCT was trained on GBV identification and risk assessment, first response (listening, validation, safety) and referral procedures. - An additional refresher training (on referral and communication skills) was also organised after enrolment. - GBV screening and risk assessment job aid was developed including 6 questions for identification and 5 for risk assessment (asking about current safety and current risk of GBV). - Prior to study implementation, EMPOWER team visited GBV referral services to select key services for the study. A referral list for study staff was developed and subsequently revised every 3 months to ensure accuracy of information. A pocket size card with referral services for GBV (e.g. shelters, counselling, legal aid services) was also developed and offered to all participants during HCT (irrespective of GBV positive disclosure). - Warm referral (study staff directly contacting support services for appointment) was also offered by study staff. - Regular debriefing sessions were given - by a GBV researcher - to study staff performing GBV screening to discuss difficult cases and implementation challenges - Staff counselling and support for vicarious trauma was organised for study staff (upon request).

**Fig. 1:**
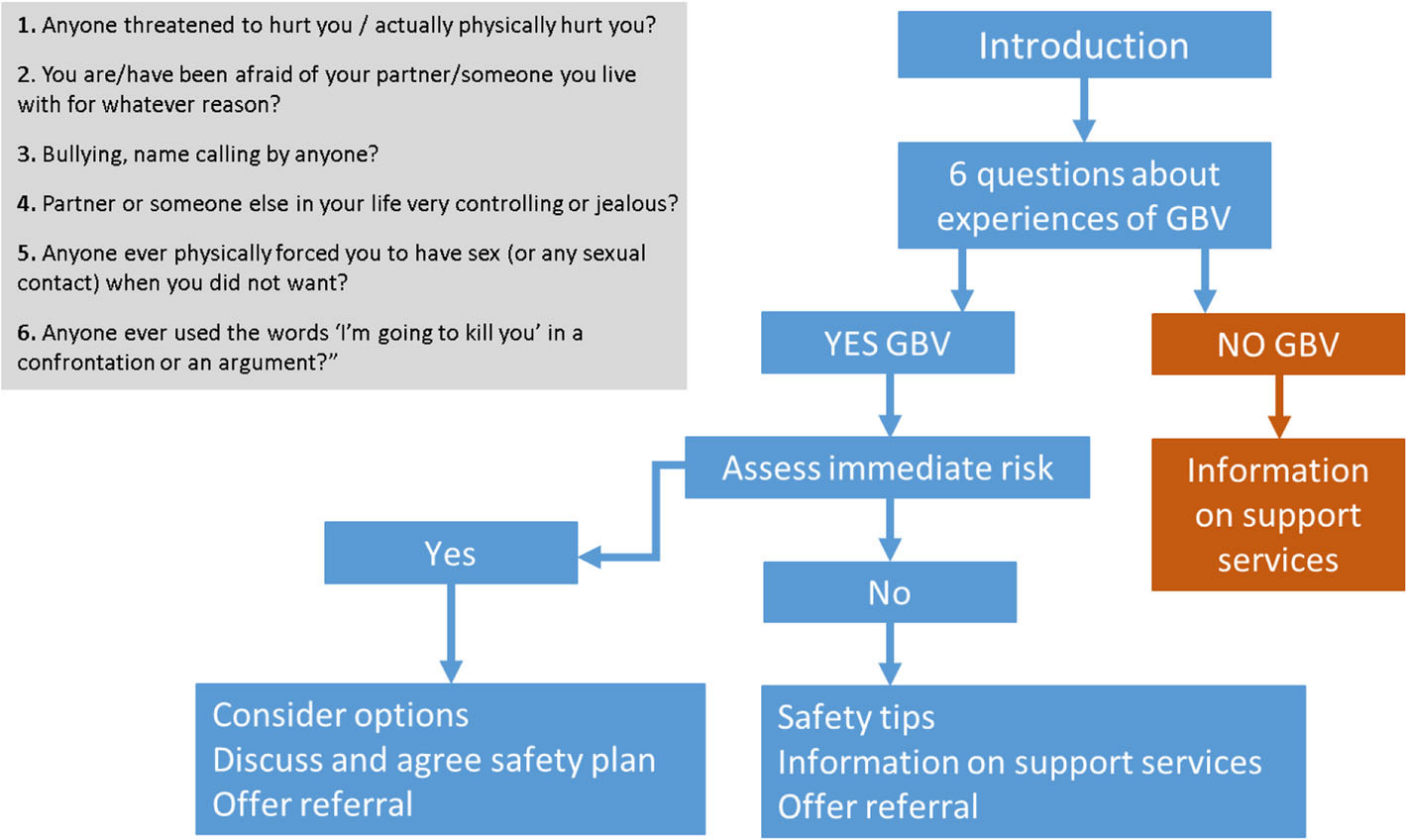
GBV screening algorithm

Consenting EMPOWER participants were counselled and tested for HIV at their first study visit, a prerequisite for PrEP initiation and refills. As part of the HIV risk assessment, participants were also screened for ever experienced (past and current) GBV by study clinical staff. GBV screening was repeated at each clinic follow-up visit (at 3 and 6 months in both sites and at 9 months in SA). The GBV screening and risk assessment questions used for the study (see Fig. 1) were adapted from World Health Organization (WHO) guidelines [28] and pre-tested with young women outside the South Africa EMPOWER cohort.

Participants found to be at immediate GBV risk of harm (at risk of severe physical or sexual violence and/or more severe mental health problems such as suicide) were referred immediately to a place of safety for further care, and counsellors discussed personalised safety plans with them. With participants who disclosed abuse but were not at immediate risk of harm, counsellors discussed referral options, organised referral if needed, and offered opportunities for safety planning. Referral to GBV support services was an integral part of the GBV screening. Participants who disclosed GBV were also followed up during adherence counselling visits within the larger EMPOWER study to also ensure GBV did not affect PrEP continuation.

### Process evaluation: data collection and sampling of participants

Between January 2017 and March 2018, we conducted a process evaluation to explore the views, experiences and practices of study participants and study clinical staff in relation to the EMPOWER GBV screening component. We used multiple data sources to assess the acceptability and feasibility of integrating GBV screening into HCT for AGYW.

We collected process data on the number of women screened, screening outcomes, and the proportion referred to further services. Field notes from observations of a random sample (*n* = 10) of integrated HCT/ GBV screening sessions (conducted by MC between January and February 2017) were included, as were notes from regular debriefings with counsellors. Data were also collected during in-depth interviews (IDIs) with 39 EMPOWER participants (SA *n* = 25; Tz *n* = 14) aged 16–24, 3 months after enrolment in the larger study. Interview participants were purposively sampled to capture a broad spread of PrEP experiences and to match the demographics of the larger study sample as far as possible, including women who became pregnant or seroconverted during the study, and women who had disclosed GBV. Semi-structured interviews were also conducted with 13 study clinical staff (SA *n* = 10; Tz *n* = 3). Table 2 describes the methods used, key questions explored and themes that guided our analysis.

**Table 2:** Data collection methods, key questions and themes that guided the analysis

Themes guiding the analysis	Key questions	Data collection methods
Process data on GBV screening	How many women were screened for GBV? How many experienced GBV? How many women were referred?	▪GBV screening outcome forms
Fidelity	How has GBV screening been delivered? How much of the GBV screening was delivered as intended? • What parts were not delivered? • Challenges to delivery?	▪In-depth interviews with study clinical staff ▪Observations of GBV screening sessions ▪Notes from debriefing meetings with counsellors
Feasibility	What were barriers and facilitators to GBV screening?	▪In-depth interviews with study participants and study clinical staff ▪Notes from debriefing meetings with counsellors
Acceptability and participants’ response	What are young women’s views and perceptions of the GBV screening? How did young women respond to GBV screening? What are study clinical staff’s perceptions of GBV screening?	▪In-depth interviews with study clinical staff and study participants
Unintended/unanticipated consequences	Were there any side-effects/unintended consequences when delivering GBV screening? (i.e. on the study participants or study clinical staff)	▪In-depth interviews with study clinical staff and with study participants ▪Notes from debriefing meetings with counsellors

Interviews were carried out by three trained researchers in the language preferred by participants, including English, isiZulu, and seSotho in SA, and Swahili in Tz. Informed consent was sought for each interview, including for audio-recording it. Interviews took place at the study clinics or a private place selected by participants. They were audio-recorded and subsequently transcribed and translated into English, where necessary.

### Data analysis

Following a thematic inductive approach [40], qualitative data were analysed using qualitative software (NVivo version 11; QSR International, Melbourne, Australia). For interview data analysis, a qualitative team developed a provisional codebook using a small selection of transcripts. Once the codebook was finalised and inductive coding of all transcripts was completed (by 2 coders), reports were then generated for specific nodes (e.g. experiences of GBV screening, views on GBV screening questions; views on integration of GBV into HCT) and summary matrices were developed to examine intersecting themes (including feasibility, acceptability and patients’ response). Two coding and analysis workshops were held to discuss interpretation of the data. Key themes from observational data (e.g. fidelity) and meeting notes were integrated with interview data as needed. Themes explored included: acceptability of and experiences with GBV screening process, feasibility of and challenges with GBV screening. Representative quotes were selected to illustrate key themes.

### Ethical approval

The study was approved by the Human Research Ethics Committee of the University of the Witwatersrand (SA), the London School of Hygiene and Tropical Medicine Ethics Committee, and the Tanzanian National Health Research Ethics Committee of the National Institute for Medical Research (Tz).

## Results

### GBV screening outcome: GBV prevalence and referrals

A total of 563 participants were screened for GBV during HCT, of which 175 (31%) reported ever having experienced GBV. Only 2 cases (in SA) were at immediate risk of harm and needed an immediate response. The remainder (*n* = 173) had experienced mostly physical and psychological violence, but were not at immediate risk for further harm. It is worth noting that sexual violence was significantly higher in Tz (62%) than in SA (28%). Only a few (SA *n* = 10; Tz *n* = 2) requested referrals to support services. Table 3 offers detailed information.

**Table 3:** GBV prevalence, types and referrals among participants screened for GBV at enrolment in the EMPOWER study

	***SA n = 482***	***Tz n = 81***	***Total n = 563***
***GBV reported at screening***^*a*^	***146*** *(30%)*	***29*** *(36%)*	***175*** *(31%)*
***Type of violence reported***			
*Sexual*	***41*** *(28%)*	***18*** *(62%)*	***59*** *(34%)*
*Psychological*	***62*** *(42%)*	***17*** *(59%)*	***79*** *(45%)*
*Physical*	***72*** *(49%)*	***9*** *(31%)*	***81*** *(46%)*
*Economic*	***6*** *(4%)*	***1*** *(3%)*	***7*** *(4%)*
*More than 1 type*	***29*** *(20%)*	***12*** *(43%)*	***41*** *(23%)*
***At risk of immediate harm***	***2*** *(1%)*	***0***	***2*** *(1%)*
***Referral requested and organised***^*a*^	***10*** *(7%)*	***2*** *(7%)*	***10*** *(6%)*

*(*^a^*reported by women and recorded in GBV Screening Outcome Forms by study counsellors)*

### Socio-demographic characteristics of study participants interviewed

In SA, most study participants interviewed qualitatively were tertiary-level students living with family or in student residences. Most Tz participants had only completed primary school, were living alone or with family members, and worked full time in food and alcohol outlets. In both sites, there was a high prevalence of GBV, with roughly half of the participants in the qualitative sub-sample reporting ever experienced any violence (Table 4).

**Table 4:** Socio-demographic characteristics of EMPOWER study participants interviewed (including GBV cases)

	South Africa	Tanzania
**STUDY PARTICIPANTS**	25	14
Age		
16–19	9	4
20–24	16	10
**Education**		
Primary school or less	0	6
Some Secondary School	3	3
All Secondary School	8	5
Some Tertiary education	14	0
**Residence**		
Alone (or in student residence)	6	5
Parents	12	3
Partner	0	1
Other relatives	7	5
**Relationship status**		
Single (not married)	25	12
Living with partner	0	1
Married	0	0
Separated or divorced	0	1
**Occupation**		
None	22	1
Full time	0	12
Part-time	3	1
**Screened positive for GBV**	13	7

### Experiences with GBV screening implementation

#### Fidelity

GBV screening was largely delivered as intended. Findings from observations of HCT sessions show that counsellors followed the GBV screening script, took time to explain confidentiality of GBV discussions, conducted risk assessments and offered information on GBV referral options - and safety tips when needed - to all study participants. However, debriefing discussions (in SA) highlighted some initial challenges. Firstly, traditional safety planning could not always be implemented, as most study participants were not living with their partners. Instead, counsellors developed additional safety tips tailored to participants’ individual situations. Secondly, immediate risk of harm was often partially assessed. However, early debriefings with the counsellors helped clarify the intended procedure for risk assessment. Both sites activated linkage to mental health services following screening, by referring cases to a social worker in an adjacent clinic for counselling (SA) or to a trained counsellor (Tz).

#### Clinical staff views on feasibility of integrating GBV screening into HCT

In SA, study counsellors found integration of GBV screening into HCT feasible, provided training and staff support was available. Many appreciated the regular GBV debriefings, which helped ‘unbottle’ their initial discomfort and emotional toll when asking about GBV, and release stress so that they did not need to ‘*take it home’*.

*‘[ … ] So if I come across that* [GBV case] *I do get support because I get debriefing [ … ] It helps a lot because what I’ve learned is, through all the experiences that I have gone through, that when you bottle up things it doesn’t help, you see, but talking about them helps a lot because like you don’t sort of like carry anything in you’.* [clinical staff, SA]

Study staff in both countries reported challenges with referral uptake with only a few participants (SA = 10; Tz = 2) accepting referral to support services, mainly for counselling and mental health services. In SA, having direct access to a social worker facilitated warm referral and follow up of referred GBV cases.

‘*Like here* [at study clinic] *we are lucky in that we got a social worker downstairs [...] you can pick up a phone and speak to someone directly* [social worker] *and say “listen, I need you to get this person”’.* [clinical staff, SA]

When referral to counselling was organised externally, some reported not attending. In Tz, traditional norms on intimate relationships and GBV (e.g. seen as a private family issue) deepened participants’ reluctance to seek help.

*‘[ … ] she does tell you* [about GBV], *but she says it’s just a story and you shouldn’t take it anywhere [ … ] Yes, it has happened to her. Ehee but she doesn’t prefer the support because ‘it’s not in our customs to report your husband*’. [clinical staff, Tz].

Because of the shyness that characterises some AGYW and pervasive cultural norms that discourage them from being sexually active, staff mentioned the added value of delivering GBV screening within the context of an adolescent and youth-friendly service.

*“[ … ] our approach like I said, the youth-friendliness, also makes it easier for them* [AGYW] *to be able to open up and talk* [about GBV]*'.* [clinical staff, SA]

Debriefings with counsellors (in SA) also revealed their uneasiness when some participants asked for advice on how to disclose PrEP use to their partners as they feared violence and rejection from their partners. Counsellors felt they lacked the necessary training or knowledge required to counsel young people on sexual relationships.

##### AGYW’s perspectives on acceptability of the GBV screening and participants’ response

Nearly all AGYW interviewed reported that they had never been asked about GBV experiences by a health provider. Almost all AGYW found GBV screening acceptable – even women who did not disclose any GBV – and reported this as a positive experience. Participants saw the GBV screening as helping them reflect on their relationships and challenging their traditional views on GBV. For some, screening offered an opportunity to discuss their GBV experience, which led to some relief.

*“Eish those questions were provoking my thoughts... [ … ], they provoke you to talk and participate, tell her* [counsellor] *and interact with her*". [GBV experience, 21 years, SA]

Many reported that discussing GBV was reassuring, as they felt that someone was willing to offer help.

*‘I felt good. [ … ] Sometimes when you tell them that I have been violated with certain kind of violence they could help you to find lawyers.’* [GBV experience, 24 years, Tz]

Irrespective of their past GBV experience, several participants reported becoming more aware and knowledgeable about GBV and gender norms through their counselling sessions.

*‘In truth I felt good because they explained to me matters that I didn’t even know and I understood.* [ … ] *That day I learned about gender violence’.* [no GBV experience, 21 years, Tz]

Even participants who reported no abuse said that the counselling provided useful information about GBV referrals.

*‘*[Screening was] *unexpected but it was exciting ‘cos I got to explain how it made me feel and how I saw it in my eyes, so it gives me an opportunity to express myself”.* [GBV experience, 18 years, SA]

Only one participant acknowledged the importance of GBV screening to help prevent HIV acquisition.

*‘You are asked on your relationship issues, gender violence... Yes, it is appropriate* [to be asked about GBV during HCT] b*ecause others get infected, they are violated, raped.’* [GBV experience, Tz]

However, a handful of participants who had experienced past violence said that being asked about abuse had been traumatic and uncomfortable, as they had never disclosed the violence before. For some, the questions felt “*too personal”* or they found it difficult to share their experiences, especially in relation to sexual violence. One participant found GBV screening hard at first, as it brought back memories of her rape experience that she wanted to forget.

‘*For me, it was just traumatising because she took me back to... you know... the past, ja, I have experienced some violence myself. So, we had to talk about that, and it was quite sad because it was something I wanted to forget, but ja*". [GBV experience, 19 years, SA]

However, she later reflected that discussing the violence had been helpful to move on. One woman shared that with time, familiarity with the counsellors made discussing GBV easier during follow-up visits.

*‘[ … ] At first, it was like for the first time and some things were personal. I … It was not easy at first but now it is easy ‘cos every time when I come, I always see them* [the counsellors] *and talk to them, ja.*" [GBV experience, 21 years, SA]

Disclosing GBV to the counsellor was reported to be emotionally easier for AGYW who had already left the abusive relationship or had received previous counselling, as this meant that they had already begun to address their emotional distress.

*‘ … it was not that hard for me because I had left the guy and I have forgiven myself and him … so it was not like... the lady brought up something, no, I didn’t have the anger and stuff."* [ GBV experience, 22 years, SA]

In the interviews, AGYW spoke about their appreciation of the study staff, depicting them as kind, friendly and caring. They found a number of qualities valuable in the staff, including the fact that they were non-judgmental and understanding, reacted positively to GBV disclosure, and did not force disclosure but accepted women’s decision not to share any painful experience.

‘S*he* [counsellor] *understands, she talks to you kindly, she doesn’t even push you to say things you don’t want to say, she just goes with the flow*". [GBV experience, 22 years, SA]

*‘They do their work whole heartedly; they aren’t discriminative in any way … I felt free’*. [no GBV experience, 19 years, Tz]

Confidentiality of GBV screening was also critical for the AGYW. They trusted the counsellors for respecting their privacy and did not fear that any disclosed information would be divulged.

‘*Because it’s private here* [at study clinic] *and I am assured that my personal answers will not go around everywhere.’* [no GBV experience, 19 years, SA]

When asked about barriers to integrating screening into routine HCT, some AGYW described the potential for women to conceal abuse out of fear of partner retaliation. Some worried that GBV screening could lead to increased risk of violence among women who were currently in abusive relationships. However, no study participants reported experiencing such unintended negative consequences.

‘*With people that are in violent situations, I don’t think … they would be comfortable with that* [GBV screening]*. [ … ] they even hide that I am in a violent relationship Because they know why “he is gonna come back for me. So let me just not touch it, you see. They won’t* [disclose].’ [no GBV experience, 18 years, SA]

## Discussion

To our knowledge, this was one of the first published studies to assess the feasibility and acceptability of integrating GBV screening into HCT services for AGYW taking PrEP. Our findings show that a third of AGYW screened for GBV prior to enrolment in the demonstration project disclosed violence, though only 10% accepted support referral. Overall, participants were amenable to being asked GBV questions, as this offered the opportunity to find emotional relief and seek help. Although the majority reported having a positive experience, for a few who reported past sexual violence screening was traumatic as it caused them to revisit past trauma. In both sites, the sensitive and empathetic approach of the staff helped mitigate such distress. In general, the delivery of GBV screening in HCT in a PrEP study proved to be acceptable, provided that the basic principles of confidentiality, staff empathy, and absence of judgment were observed. However, uptake of linkage to further care remained low in both sites.

Crucial requirements for delivering GBV screening included continuous training on basic response and support of staff. The study reaffirms the evidence that experiential learning – through regular debriefings – is crucial for maintaining staff knowledge, increasing self-efficacy and shaping providers’ practices [41, 42]. Mentoring programmes and group discussions of difficult GBV cases with more senior clinical staff could help mitigate challenges. However, integrating such practice into busy and overstretched HIV clinics may be difficult [23, 43]. Health managers should ensure clear protocols and care pathways for discussing GBV are in place, and a supportive environment for clinical staff to overcome such barriers.

Although having a referral system is critical when screening for GBV in health services [26], it is worth acknowledging the limited uptake of referral services by participants in this study. This is in line with current evidence showing complex reasons for such disparities (between formal referral and uptake), including low trust in the quality of formal services, cultural norms (e.G. *stigma*, fear of family repercussions), time and financial constraints [44–46]. More research is clearly warranted on referral preferences among AGYW, however. In both sites, having direct access to a social worker or trained counsellor facilitated GBV referrals uptake, as also evidenced elsewhere [41, 47, 48]. Some women still preferred to talk only to the study staff and were wary and distrustful of external services. Though in practice, having on-site support may not be a viable option in all public clinics, alternative linkages to quality support services should be put in place before integrating GBV screening into HCT, particularly for psychological care. However, such services are often unavailable, underdeveloped, and stigmatized, although they are urgently needed for this age group [49].

Low relationship power in itself is an important predictor of HIV risk and IPV among AGYW [50], and AGYW may have little power in their intimate relationships to elicit support for PrEP use from partners. Our study has shown the need for training PrEP counsellors to also discuss power relations within sexual partnerships and gender inequality more broadly with AGYW. Additional investigation into how training could also address communication skills when discussing PrEP adherence with this group is critical. Ideally, PrEP counselling should offer a safe space for AGYW to discuss relationship concerns and GBV experiences that could affect PrEP engagement.

Our findings also suggest the importance of having a youth-friendly approach. Kindness, privacy, time, empathy and lack of judgement were all reported as factors facilitating GBV disclosure. Evidence from sub-Saharan Africa shows that some of these elements are poorly implemented in public clinics due to lack of youth-friendly training among staff, no dedicated space for young people, and low respect for confidentiality – all of which hinder the potential integration of violence identification for adolescents [51–53]. While the GBV screening component in EMPOWER was relatively simple to implement, the importance of training staff to be sensitive, non-judgemental, and to provide confidential services should not be underestimated [34, 54].

Lastly, when conducting GBV screening for AGYW, we have learned the importance of going beyond IPV and dating violence to also focus on non-partner sexual violence, as recommended by regional studies [11]. In our study, many participants (in both sites) were unmarried and not living with partners, which affected how safety could be practically secured for this group. Further research is critical to explore preferences and adapt safety plans and tips, and tailor them to the specific social circumstances of the target group.

### Limitations

The GBV screening evaluation was conducted in the context of a demonstration study rather than in routine HIV services, at a time when PrEP was not yet standard of care. However, the information in this article offers useful insights into factors affecting acceptability and feasibility for programme designers seeking to integrate GBV screening into HIV prevention and care settings for AGYW. Although most interviews were conducted by researchers not involved in the study, it is possible that participants viewed them as study implementers and gave socially desirable responses that showed the screening in a positive light. Furthermore, observing counselling sessions may have made counsellors behave differently because they knew they were being observed. Lastly, differences in samples from the two countries (particularly as it relates to their household configurations) might have affected some of the conclusions drawn around safety.

## Conclusion

Most stakeholders found integrated GBV screening in an HCT and PrEP context acceptable and feasible. Future GBV screening interventions for AGYW within HCT should consider key principles such as respect for confidentiality, a youth-friendly and non-judgmental environment, and ensuring safety. A functioning referral network, experiential learning and mentoring were critical for sustaining GBV screening and should not be underestimated in future similar interventions.

## Data Availability

The qualitative dataset generated during the study is not publicly available due to the sensitive nature of the data collected. However, the author will gladly provide any supporting information upon request.

## Abbreviations

AFYS: Adolescent and youth-friendly services
AGYW: Adolescent girls and young women
IPV: Intimate partner violence
GBV: Gender-based violence
HCT: HIV counselling and testing
PrEP: Pre-exposure prophylaxis
SA: South Africa
Tz: Tanzania
